# Thermoreversibly Cross-Linked EPM Rubber Nanocomposites with Carbon Nanotubes

**DOI:** 10.3390/nano8020058

**Published:** 2018-01-23

**Authors:** Lorenzo Massimo Polgar, Francesco Criscitiello, Machiel van Essen, Rodrigo Araya-Hermosilla, Nicola Migliore, Mattia Lenti, Patrizio Raffa, Francesco Picchioni, Andrea Pucci

**Affiliations:** 1 Department of Chemical Engineering, University of Groningen, Nijenborgh 4, 9747 AG Groningen, The Netherlands; L.m.polgar@rug.nl (L.M.P.); machielvanessen@live.nl (M.v.E.); r.a.araya.hermosilla@rug.nl (R.A.-H.); nicola_migliore@hotmail.it (N.M.); mattia.lenti@ymail.com (M.L.); p.raffa@rug.nl (P.R.); 2 Dutch Polymer Institute (DPI), P.O. Box 902, 5600 AX Eindhoven, The Netherlands; 3 Department of Chemistry and Industrial Chemistry, University of Pisa, Via Moruzzi 13, I-56124 Pisa, Italy; francrisci86@gmail.com (F.C.); andrea.pucci@unipi.it (A.P.)

**Keywords:** strain sensor, rubber nanocomposite, thermoreversible cross-linking, Joule effect, crack-healing

## Abstract

Conductive rubber nanocomposites were prepared by dispersing conductive nanotubes (CNT) in thermoreversibly cross-linked ethylene propylene rubbers grafted with furan groups (EPM-g-furan) rubbers. Their features were studied with a strong focus on conductive and mechanical properties relevant for strain-sensor applications. The Diels-Alder chemistry used for thermoreversible cross-linking allows for the preparation of fully recyclable, homogeneous, and conductive nanocomposites. CNT modified with compatible furan groups provided nanocomposites with a relatively large tensile strength and small elongation at break. High and low sensitivity deformation experiments of nanocomposites with 5 wt % CNT (at the percolation threshold) displayed an initially linear sensitivity to deformation. Notably, only fresh samples displayed a linear response of their electrical resistivity to deformations as the resistance variation collapsed already after one cycle of elongation. Notwithstanding this mediocre performance as a strain sensor, the advantages of using thermoreversible chemistry in a conductive rubber nanocomposite were highlighted by demonstrating crack-healing by welding due to the joule effect on the surface and the bulk of the material. This will open up new technological opportunities for the design of novel strain-sensors based on recyclable rubbers.

## 1. Introduction

“Smart rubbers” are defined as elastomeric materials that respond to external stimuli through a macroscopic output in which the energy of the stimulus is transduced appropriately as a function of external interference [1]. Polymer or rubber nanocomposites have gained scientific and technological interest because they often exhibit enhanced or novel properties compared with the neat polymer or conventional composites at the same filler loading. The incorporation of carbon nanotubes (CNT) into such polymer matrices yields nanocomposites with high strength and electrical conductivity [2]. These nanocomposites have found their way into a variety of applications, especially in the field of electrically-conductive plastic networks [3,4,5]. 

Thermoplastic elastomers (TPE) are attractive supporting materials for CNT because they are easily processed and fabricated into solid-state forms, such as thin films that are often required for applications. Such TPE/CNT nanocomposites can be prepared by either melt blending or in situ polymerization [6,7,8], but solution mixing is the most effective process to produce them at a small sample level. In this case a solvent is used to disperse CNT, generally attained by ultrasonication—recognizing that significant damage of their structures as well as shortening occur thus limiting the full potential of CNTs as additives in polymer nanocomposites—and/or opportune amounts of surfactants to produce a metastable suspension of nanotubes. The polymer, dissolved separately in the same solvent, is then added to the mixture. The final nanocomposite is obtained after solvent evaporation at reduced time by spin-coating the suspension, thereby reducing the typical CNT re-aggregation. 

TPE nanocomposites containing CNT have received considerable attention in the literature due to the development of stretchable resistivity-strain sensors for detecting dangerous deformations and vibrations of mechanical parts in many fields of science and engineering [9,10,11,12]. In these nanocomposites, the applied strain induces carbon nanotube displacement/sliding on the microscale, as well as tensile deformation applied locally to individual CNT. These responses give rise to piezo resistive behavior as applied tensile strains yield measurable changes in electrical resistivity across the composite length. Nanocomposites in which 0.01–5 wt % of CNT (the corresponding percolation threshold) were dispersed in a polymeric matrix of styrene-butadiene-styrene rubber (SBS) [13], polymethyl methacrylate (PMMA) [14], polystyrene (PS) [15], thermoplastic polyurethane (TPU) [12,16,17,18] or combinations thereof [19] all display a similar behavior as their surface resistivity was correlated with the applied strains and observed to increase with increasing tensile strain. This behavior was addressed to the reduction in conductive network density and increase in inter-tube distances induced by deformation. 

**Figure 1:**
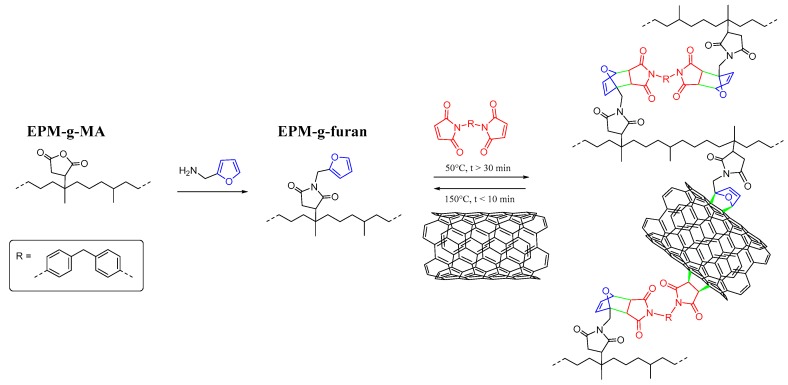
Furan functionalization and bismaleimide (BM) cross-linking of EPM-g-furan and the integration of CNT fillers via covalent interactions in the thermoreversibly cross-linked network of the nanocomposite.

Ethylene propylene diene rubbers (EPDM) are one of the most frequently used materials in such TPE and can be found in window profiles, automotive, and roofing applications. Cross-linked EPDM rubbers are ideal candidates for low-cost elastomeric-based stress-strain sensors containing CNT as they display relatively high moduli, strengths, and elasticities, and are renowned for their good weather, temperature, chemical, ozone, and stress cracking resistance [20]. Cross-linking the rubber matrix also helps to overcome the general complications associated with the utilization of CNT and the strong van der Waals interactions between individual nanotubes that make achieving a uniformly dispersed composite at the nanoscale difficult. Unfortunately, the excellent properties of these typically sulfur vulcanized and peroxide cured EPDM rubber compounds are associated with the practical impossibility of reprocessing them after their product life. A recently developed alternative to these conventional, irreversible cross-linking techniques is found in thermoreversible cross-linking via Diels-Alder (DA) chemistry [21,22,23]. A good example is found with bismaleimide (BM) cross-linking of furan-functionalized EPM rubbers [21] as the resulting covalent cross-links yield a material with properties similar to those of conventionally cross-linked EPDM gum rubbers that are retained upon reprocessing. This material would therefore be an excellent candidate for the preparation of nanocomposites containing well-dispersed carbon nanotubes. While the system itself allows for the preparation of a fully cradle-to-cradle recyclable, conductive nanocomposite, the functional groups on the polymer backbone allow for various interactions with (defects in the) CNT [24] that may yield durability for subsequent cycles of measurements (Figure 1).

The goal of this work was to study the material properties (with a strong focus on conductive and mechanical properties that are relevant for strain-sensor applications) and reprocessability of nanocomposites based on thermoreversibly cross-linked EPM rubbers and CNT. This is done in the context of developing materials for strain-sensor applications. First, the effect of adding various amounts of CNT to thermoreversibly cross-linked EPM rubbers on their dispersion throughout the rubber matrix and the material properties of the resulting nanocomposites is studied and their ultimate use as strain-sensors evaluated. Only multi-walled CNT are used for this purpose as it was found that these yield nanocomposites with a higher electrical conductivity and piezoresistive sensitivity than single-walled CNT as a result of their metallic character [18,19]. Secondly, both the rubber matrix and the CNT are chemically functionalized to stimulate the formation of primary or secondary interactions between them. The effects of such interactions on the dispersion of the CNT is studied as this may improve the compatibility of both components and thereby enhance the material properties of the resulting nanocomposite. This may also affect the material properties of the rubber nanocomposites with respect to their application as strain sensor. Finally, the advantages of using thermoreversible chemistry in a conductive rubber nanocomposite are highlighted by demonstrating crack-healing by welding due to the joule effect on the surface and the bulk of the material.

## 2. Materials and Methods

### 2.1. Materials

A maleated EPM (EPM-g-MA, Keltan DE5005, 49 wt % ethylene, 2.1 wt % MA, *M*_n_ = 50 kg/mol, polydispersity index = 2.0) were kindly provided by ARLANXEO Performance Elastomers. Furfurylamine (FFA, Sigma-Aldrich, St. Louis, MO, USA, ≥99%) was freshly distillated. Multi-walled carbon nanotubes (CNT, Sigma-Aldrich, diameter × L 6–9 nm × 5 µm, >95% (carbon)) were used as additive and cross-linking agent. 1,1′-methylenedi-4,1-phenylene)bismaleimide (BM, Sigma-Aldrich, ≥97%) and dicumyl peroxide (DCP, Sigma-Aldrich, 98%) were used as reversible and irreversible cross-linking agents, respectively. 3-azido-1-propanamine (90%), octadecyl-1-(3,5-di-tert-butyl-4-hydroxyphenyl) propionate (anti-oxidant, 99%), 1-methyl-2-pyrrolidinone (NMP, 99.5%), tetrahydrofuran (THF, >99.9%), decahydro naphthalene (decalin, mixture of *cis* + *trans*, >98%) and acetone (>99.5%) were all bought from Sigma-Aldrich and used as received.

### 2.2. Methods

#### 2.2.1. Furan-Functionalization of EPM-g-MA

Prior to the reaction, EPM-g-MA was dried in a vacuum oven for 1 h at 175 °C to convert the present diacids into anhydrides [21]. The EPM-g-MA precursor was then converted into EPM-g-furan using FFA according to a reported procedure [21]. 

#### 2.2.2. Amine Modification of CNT

An amount of 3.00 g CNT was dispersed in 240 mL NMP by sonication for 30 min. Then 11.33 g 3-azido-1-propanamine was added and refluxed at 160 °C for 24 h under a N_2_ atmosphere. The resulting solution was diluted with 250 mL acetone and centrifuged for 15 min. The solvents were removed to recover the modified CNT. Then 480 mL acetone was added to the CNTs and the suspension was sonicated for 30 min. Again, the mixture was centrifuged at 4500 rpm for 15 min and the solvent was removed. This washing cycle was repeated 5 times. Finally, the product was dried in an oven at 70 °C for 2 days to yield 2.57 g of amine modified CNT. The amine-modification of CNT was analyzed by elemental analysis (EA: 2.64 wt % N, 92.9 wt % C, and 0.65 wt % H). The modified CNT display a functionalization degree of 0.94 mmol/g, which is comparable to values found in literature in an acceptable range (1 added in 10 to 100 carbon atoms) [2].

#### 2.2.3. Solution Mixing and Cross-Linking of Nanocomposites

Typically, 5.0 g of EPM-g-furan rubber was dissolved in 50 mL THF. Meanwhile, 0.5 to 10 wt % of CNT (with respect to EPM-g-furan) was exfoliated by suspending in 50 mL THF and sonicating for 30 min. Both solutions were then mixed and homogenized by stirring for 15 min and sonicating for 30 min. Then 0.5 molar equivalent (based on the furan content of EPM-g-furan) of cross-linking agent (BM or DCP) 1000 ppm phenolic anti-oxidant were dissolved in approximately 2 mL of THF and added to the mixture before refluxing it for 24 h. After mixing all components, the solvent was removed and the remaining product was dried in an oven at 50 °C for 24 h. Finally, the resulting nanocomposite was compression molded at 150 °C and 100 bar for 30 min and thermally annealed in a 50 °C oven for 3 days. Samples were reprocessed by grinding them into a ball mill at −195 °C and compression molding the resulting powder into new sample bars at 150 °C and 100 bar for 30 min and thermally annealing them in a 50 °C oven for 3 days.

### 2.3. Characterization

The conversion of EPM-g-MA to EPM-g-furan was followed by Fourier Transform Infrared spectroscopy (FT-IR) and EA. FT-IR spectra were recorded on a Perkin-Elmer Spectrum 2000 (Perkin Elmer, Waltham, MA, USA). Rubber films with a thickness of 0.1 mm were compression molded at 150 °C and 100 bar for 30 min, thermally annealed to ensure maximum DA cross-linking and measured in a KBr tablet holder. Measurements were performed over a spectral range from 4000 to 600 cm^−1^ at a resolution of 4 cm^−1^, co-averaging 32 scans. Deconvolution was used to quantify the areas under the individual FT-IR peaks (*R*^2^ > 0.95). The differences in relative peak areas were used to calculate the reaction conversion. The methyl rocking vibration peak at 723 cm^−1^ was used as an internal reference, as it originates from the EPM backbone and is not affected by chemical modification. The decrease of the absorbance of the C=O symmetrical stretch vibration of the anhydride groups at 1856 cm^−1^ was used to calculate the conversion of the reaction from EPM-g-MA to EPM-g-furan, according to a reported procedure [21]. The decrease of the characteristic C–O–C symmetrical stretch vibration of the furan groups at 1013 cm^−1^ was used to determine the conversion of the cross-linking reaction in the same way. EA for the elements N, C and H was performed on a Euro EA elemental analyzer. The nitrogen content was related to the furan-functionalization according to a reported procedure [21] and to the amine functionalization of CNT as no nitrogen is present in the non-modified CNT.

Gel Permeation Chromatography (GPC) was performed using triple detection with refractive index, viscosity, and light scattering detectors, i.e., a Viscotek Ralls detector (Malvern Instruments Ltd., Malvern, UK), a Viscotek Viscometer Model H502 (Malvern Instruments Ltd., Malvern, UK) and a Shodex RI-71 Refractive Index detector (Showa Denko Europe GmbH, Munich, Germany), respectively. The separation was carried out using a guard column (PL-gel 5 µm Guard, 50 mm) and two columns (PL-gel 5 µm MIXED-C, 300 mm) from Agilent Technologies (Amstelveen, The Netherlands) at 30 °C. THF 99+%, stabilized with butylated hydroxytoluene, was used as the eluent at a flow rate of 1.0 mL/min. The samples (~2 mg/mL) were filtered over a 0.45 µm PTFE filter prior to injection. Four GPC measurements were performed on each sample. Data acquisition and calculations were performed using Viscotek OmniSec software version 4.6.1 (Malvern Instruments Ltd., Malvern, UK), using a refractive index increment (d_n_/d_c_) of 0.052. Molecular weights were determined using a universal calibration curve, generated from narrow polydispersity polystyrene standards (Agilent and Polymer Laboratories, Santa Clara, CA 95051, USA). 

Equilibrium swelling experiments were performed in decalin at room temperature. The rubber sample (approximately 500 mg) was weighed in 20 mL vials (*W*_0_) and immersed in 15 mL solvent until equilibrium swelling was reached (3 days). The sample was then weighed after removing the solvent on the surface with a tissue (*W*_1_) and was dried in a vacuum oven at 110 °C until a constant weight was reached (W_2_). The gel content of the gum rubber samples is defined as (*W*_2_/*W*_0_) 100%. The apparent cross-link density [XLD] was calculated from W_1_ and W_2_ using the Flory-Rehner Equation (1) [25,26,27]. It is noted that the Flory-Rehner equation is only applicable for homogeneous rubber samples with difunctional cross-links, whereas the samples in this study are rubber nanocomposites containing up to 10 wt % of CNT. The calculated values therefore only represent apparent cross-link densities.

(1) $$\left[\mathrm{XLD}\right]=\;\frac{\ln\left(1-V_{R}\right)+V_{R}+\chi V_{R}^{2}}{2V_{S}\left(0.5V_{R}-V_{R}^{\frac{1}{3}}\right)}\;\mathrm{with}\;V_{R}=\;\frac{W_{2}}{W_{2}+\left(W_{1}-W_{2}\right)\cdot \frac{\rho_{\mathrm{EPM}-g-\mathrm{furan}}}{\rho_{\mathrm{decalin}}}}$$

*V*_R_ Volume fraction of rubber in swollen sample.
*V*_S_ Molar volume of solvent (decalin: 154 mL/mol at room temperature).
*χ* Flory-Huggins interaction parameter (decalin-EPDM: 0.121 + 0.278*V*_R_) [28].
*ρ* Density (0.860 g/mL for EPM-g-furan and 0.896 g/mL for decalin).

Thermographic analysis (TGA) was performed using a Mettler Toledo TGA/SDTA851e (Mettler Toledo, Columbus, OH, USA), connected to an auto robot TS0801RO with a Mettler Toledo TS0800GC1 Gas Control unit. The samples were heated from 20 °C to 600 °C at 10 °C per min under nitrogen to pyrolyze the rubber part of the residue, while leaving the CNT unaffected. 

The surfaces of the nanocomposites were characterized by scanning electron microscopy (SEM) imaging using a Philips XL30 Environmental SEM FEG instrument (Philips, Amsterdam, The Netherlands). Samples were prepared by cryogenic fracture in order to create a surface with exfoliated CNTs.

X-ray photoelectron spectroscopy (XPS) was performed on a SSX-100 spectrometer (Surface Science Instrument, Fisons plc, Ipswich, Suffolk, UK) equipped with a monochromatic Al Kα X-ray source (*h*_v_ = 1486.6 eV) that operates at a base pressure of 3 × 10^−10^ mbar. The CNTs samples were prepared by re-suspending in toluene and drop-casting on golden substrates. After evaporation of the solvent, the samples were transferred into an ultra-high vacuum system.

Tensile strength (*T*_b_) and elongation at break (*E*_b_) were measured on an Instron 5565 (Instron, High Wycombe, UK) with a clamp length of 15 mm, according to the ASTM D412 standard. A displacement rate of 500 ± 50 mm/min was applied. For each measurement 10 samples were tested and the two outliers were excluded to calculate the averages. The median stress-strain curves are shown in the figures. Cyclic hysteresis tests were performed on the same instrument with a clamp length of ±3 cm. Samples were subjected to 5 cycles of 5%, 10%, 15%, and 20% strain with a strain rate of 10% of the sample length per minute. 

The percolation threshold was determined by measuring the conductive behavior as function of the CNT loading. The electrical resistance of each sample was measured 3 times at various places of the sample at a length of ±1 cm with a Keithley multimeter (model 2010, Keithley Instruments, Cleveland, OH, USA).

**Figure 2:**
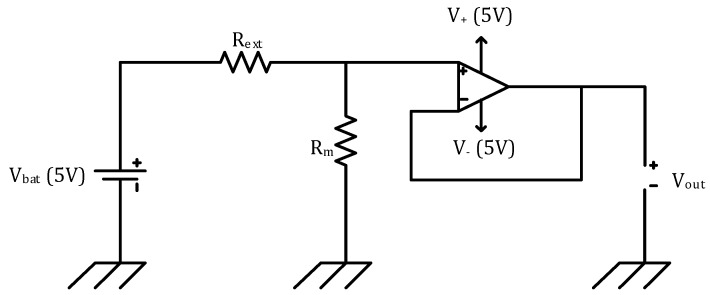
Schematic illustration of the circuit used for determining the sample resistance under strain.

Low sensitivity deformation of the nanocomposites was tested by measuring their cyclic conductive behavior under strain on an Instron 4464 (Instron, High Wycombe, UK) with a clamp length of 3 cm. Samples were subjected to 5 cycles of 5%, 10%, 15% and 20% strain with a strain rate of 10% of the sample length per minute. The resistance (*R*_m_ = (*R*_ext_ × *V*_out_)/(*V*_in_ − *V*_out_)) was digitally monitored according to a specific circuit (Figure 2) where *R*_m_ is the sample resistance and *R*_ext_ is an external resistance of equal magnitude.

High sensitivity deformation testing of the CNT filled rubber nanocomposites was performed at around the percolation threshold. The conductive behavior of the nanocomposites under strain was measured on a Tinius Olsen H25KT tensile tester (Tinius Olsen TMC, Horsham, PA, USA) with a clamp length of 1 cm. Nanocomposite sample bars were clamped in between copper sheets stretched manually in steps of ±2 mm and holding them in position for 30 s to measure the resistance with a Gossen Metrawatt Metrahit 18S multimeter (GMC-I Messtechnik GmbH, Nürnberg, Germany). Three deformation cycles were performed for each sample after at least 24 h from deformation, thus allowing the complete elastic recovery of the specimen.

**Figure 3:**
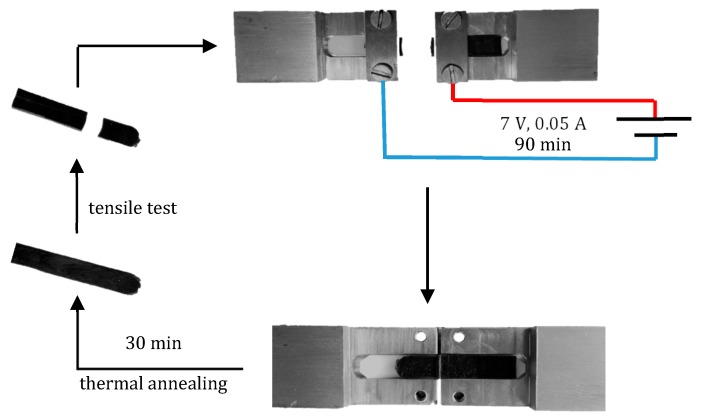
Illustration of the practical procedure to perform welding tests using a home-made device and exposing the freshly cut sample bar to a source potential (I).

The Joule effect was visualized by collecting thermographic images with a Fluke Ti10 IR Fusion Technology camera (Fluke Corporation, Everett, WA, USA) at steady state heat generation. The thereby enabled crack-healing by welding of the nanocomposites was demonstrated via a scratch test and by re-annealing broken tensile test samples. Scratch tests were performed by polymer solution casting of a nanocomposite film on a glass microscope slide, making a microscopic scratch on the surface with a scalpel. The film was then exposed to a potential source (7 V and 0.05 A) for 30 min by clamping the metal wires on the edges of the film in between the glass microscope slide substrate and another one covering it. The slow disappearance of the scratch was observed using an Zeiss Axioskop with HCS MX5 framegrabber (Zeiss, Oberkochen, Germany). Current induced welding of the bulk of the rubber nanocomposites was performed by cutting sample bars in half and pushing the freshly cut surfaces of the two halves together in a home-made device (Figure 3). The sample in the device was exposed to a potential source (7 V and 0.05 A) for 90 min. The welded samples were left at room temperature for 30 min before re-examining them. 

## 3. Results and Discussion

### 3.1. The Reinforcing Effect of CNT in Rubber Nanocomposites

#### 3.1.1. Chemical Characterization

The conversion of EPM-g-MA into EPM-g-furan was successful with high yields (>95%) according to FT-IR and EA [21]. Differential scanning calorimetry of the (non-cross-linked and cross-linked) rubbers showed a *T*_g_ of approximately −61 °C with the addition of CNT resulting in an expected increase in *T*_g_ of up to merely 3 °C for a 10 wt % CNT loading (data not shown for brevity) as a result of the decrease in segmental mobility of the polymer chains [20]. TGA thermograms all display a strong weight loss around 450 °C attributed to the degradation of the polymer matrix. This transition temperature is ~10 °C higher for samples loaded with CNT, which is attributed to the scavenging properties of graphitic fillers [27]. The amount of residue remaining at the end of analysis (600 °C) corresponds to the CNT content expressed in percentage by weight (Table 1). These values correspond to the amount of loaded CNT, indicating that the developed experimental methodology allows a complete transfer of the entire graphitic mass into the polymer matrix. The gel content of the EPM-g-furan nanocomposites is systematically larger than that of EPM-g-MA at the same CNT loading. This suggests some special interaction between the CNT surface and the furan groups that are grafted on the polymeric backbone [2,24,29]. The relatively high gel contents of the BM cross-link samples indicate that all chains are part of the rubber network. The systematic increase in the apparent cross-link density with the CNT loading of all BM cross-linked rubbers indicates that the CNT fillers participate in the formed rubber network. 

#### 3.1.2. Morphological Characterization

**Figure 4:**
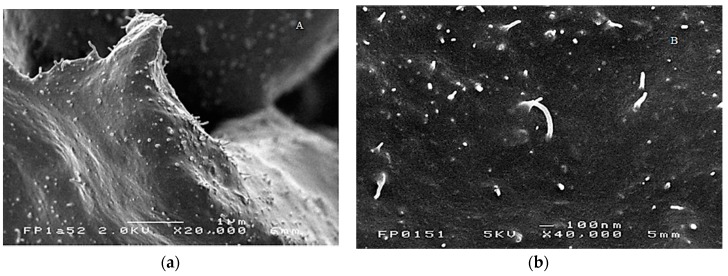
Scanning electron microscopy (SEM) micrograph of EPM-g-furan/CNT nanocomposites with (**a**) 7 wt % and (**b**) 10 wt % CNT loading.

SEM micrographs of the fractured surface of the CNT filled polymeric matrices display CNT as white filaments (Figure 4). From the micrographs, it is evident that the CNT are distributed in homogeneously dispersed bundles throughout the polymer matrix. The relatively large diameter of these bundles (30–50 nm with respect to 6–9 nm for single CNT) may also imply that the surface of the CNT is covered with a layer of polymer as has previously been observed for polycarbonate/CNT composites [3].

#### 3.1.3. Tensile Properties of BM Cross-Linked EPM-g-Furan Nanocomposites with CNT

**Figure 5:**
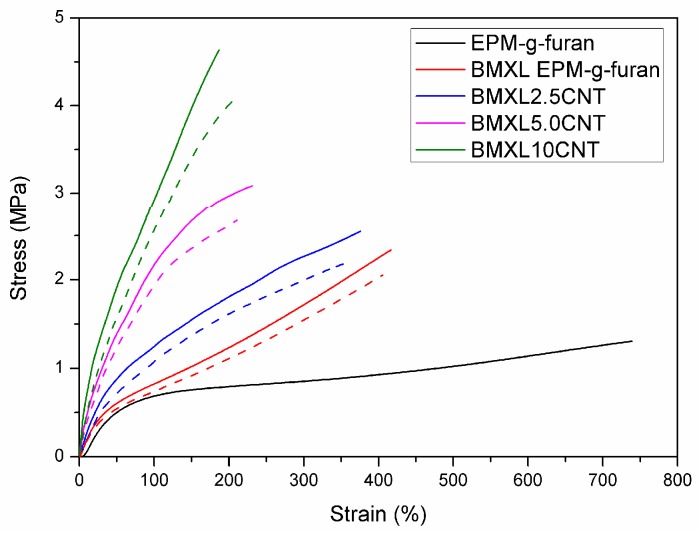
Median stress-strain curves of (BM cross-linked) EPM-g-furan/CNT nanocomposites with various CNT loading before (solid lines) and after reprocessing (dashed lines).

The stress-strain curve of EPM-g-furan is typical for a non-cross-linked rubber with an extremely large *E*_b_ and a very low *T*_b_ (Figure 5). As expected, the *T*_b_ increases and the *E*_b_ break decreases upon BM cross-linking. The Young’s modulus and *T*_b_ evidently increase and the *E*_b_ decreases upon the addition of CNT as reinforcing additives to both non-cross-linked EPM-g-furan (stress-strain curves not shown for brevity) and BM cross-linked EPM-g-furan. This means that the CNT are successfully incorporated into the rubber matrix, i.e., they display their characteristic toughening and reinforcing ability. Finally, reprocessing of the BM cross-linked EPM-g-furan sample bars yielded new coherent samples (impossible for the peroxide cured reference samples) with material properties that are similar (approximately 90% retention of properties) to those of the original samples. This is evidence that the addition of CNT does not significantly affect the reprocessability of the BM cross-linked EPM-g-furan rubbers.

**Figure 6:**
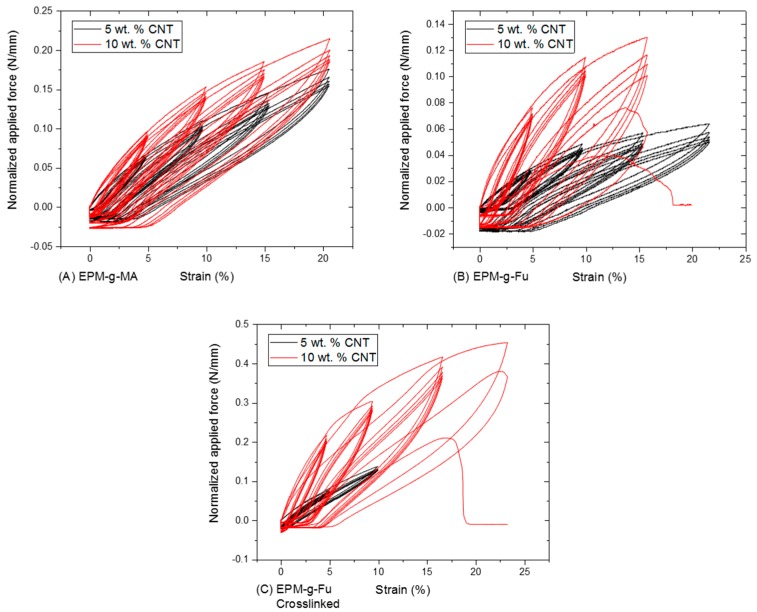
Cyclic tensile test results of CNT nanocomposites with (**A**) EPM-g-MA, (**B**)EPM-g-furan and (**C**) BM cross-linked EPM-g-furan (right) containing 5 and 10 wt % of CNT.

All samples used for cyclic tensile tests display elastic hysteresis (Figure 6). Deforming the composite by loading and unloading the material with force therefore results in an internal deformation and rearrangement of the Amatrix and dispersion and stabilization of the CNT [24]. At the first stage of extension a relatively large amount of force is required to overcome any physical interactions and to align the polymer chains and CNT. Unloading the material results in a reversed behavior as initially the applied force per decrease in elongation decreases, evidencing the retraction and energetically favored rearrangement of the polymer chains. These internal rearrangements and deformations cause dissipation of energy for every tensile cycle. Less force is therefore required to reach the same level of elongation when applying multiple cycles. The softening effect (decrease in toughness) is decreasingly visible for every cycle and increases with the exerted extension. This indicates that the CNT in the matrix gradually disconnect from each other, making the nanocomposites possibly suitable for sensor applications. Toughening of the samples is directly correlated to the CNT content and is especially evident for the BM cross-linked samples.

#### 3.1.4. Tensile Properties of Rubber Nanocomposites with Various (Modified) CNT

**Figure A1:**
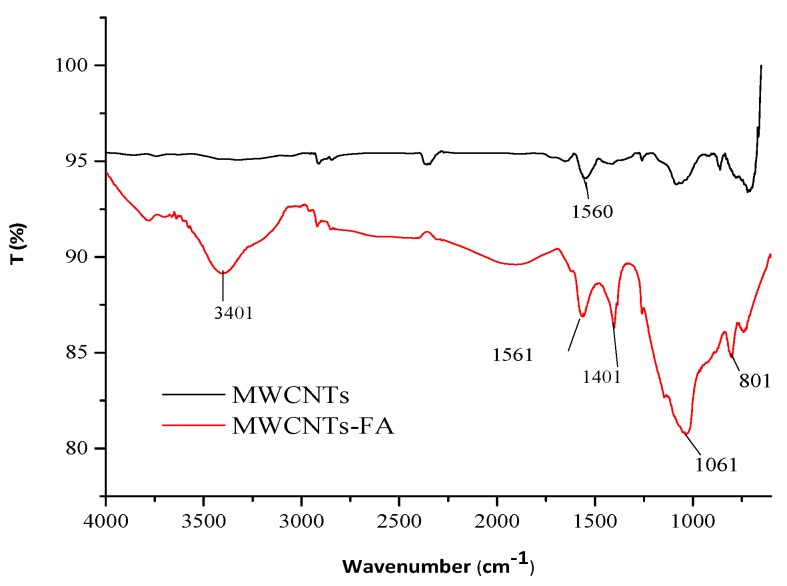
Comparison between the FT-IR spectra of pristine carbon nanotubes (black) and functionalized carbon nanotubes (red).

**Figure A2:**
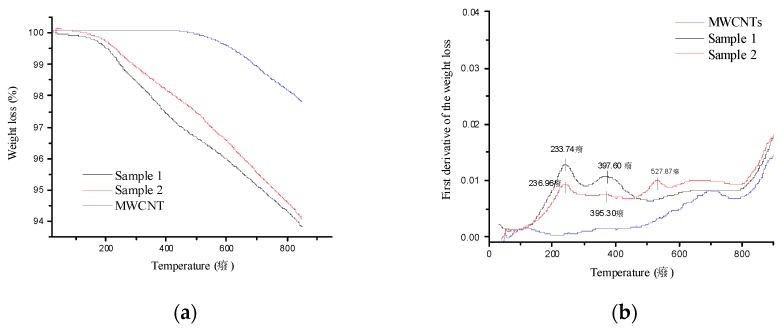
(**a**) TGA analysis under nitrogen atmosphere of pristine MWCNTs (blue) and two different batches of MWCNT -FAs (red and black) FA obtained under the same conditions of functionalization and (**b**) the first derivative of these curves.

**Figure A3:**
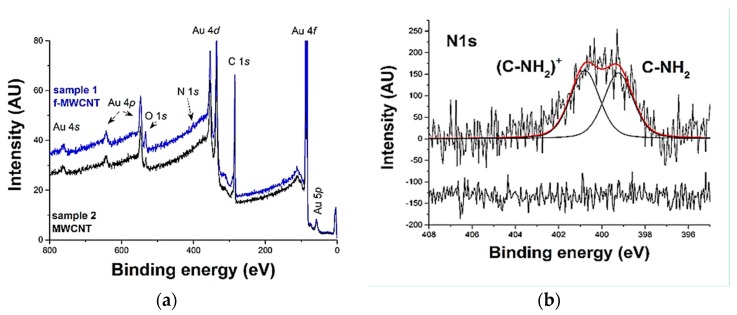
(**a**) XPS spectra of non-functionalized (black/down) and FFA functionalized (blue/up) CNT and (**b**) the first derivative of those spectra in the region relative to the peak photoemission on the N 1s orbital at around 400 eV. The signal was analyzed by deconvolution operated by the instrument software.

**Figure A4:**
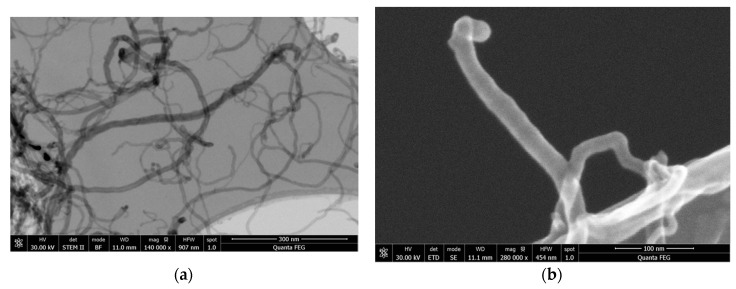
SEM images at of a FFA functionalized CNT, with an increase in magnification in going from (**a**) to (**b**), display that both the length (500 nm) and the diameter (20 nm) are still in agreement with the dimensions assessed by the producer.

The CNT loading appears to directly correlate to an increase in *T*_b_ and a decrease in EB for both the regular and the modified CNT (Table 2). The amine modification of CNT was also successful (2 wt % of FFA attached) according to FT-IR (Figure A1), TGA (Figure A2), XPS (Figure A3) and SEM (Figure A4). The amino functionalized CNTs appear to result in nanocomposites with a larger *T*_b_ and smaller *E*_b_ than with the original CNT, possibly due to the formation of effective interactions with the polymer matrix. Considering in a similar the modifications provided by the modified CNT to the mechanical properties of EPM-g-MA and EPM-g-furan, these interactions might be addressed to secondary interactions only. The amino functionalities could indeed react with EPM-g-MA thus generating amide or imide covalent linkages, which would be even more effective in mechanical properties modifications [30]. 

The small difference in material properties between the filled EPM-g-MA and EPM-g-furan samples may imply the presence of some interactions between the furan groups grafted onto the polymer backbone and the CNT. Similar interactions between polymer-linked furan groups and CNT have been described in the literature [24,31,32,33]. The effect of the addition of CNT on the material properties of the BM cross-linked EPM-g-furan is also more evident than for the non-cross-linked precursors. This well-known effect is also attributed to the interactions between the CNT and the polymer chains in the rubber matrix [9,34]. These interactions evidently increase the effective degree of cross-linking (Table 1), resulting in the expected effect on the tensile properties. To investigate such interactions, BM and (exfoliated) CNT were mixed in THF, refluxed for 1 day, filtered, washed, and dried in an oven. It appeared that approximately 0.05 mmol BM was absorbed per g CNT. These (reversible) interactions between furan or BM and CNT are even more prevalent in the nanocomposites with modified CNT fillers as is evident from the larger *T*_b_ and smaller *E*_b_ of all rubber samples with the same filler loading.

### 3.2. Conductive Behaviour of BM Cross-Linked Rubber Nanocomposites

#### 3.2.1. Percolation Threshold

**Figure 7:**
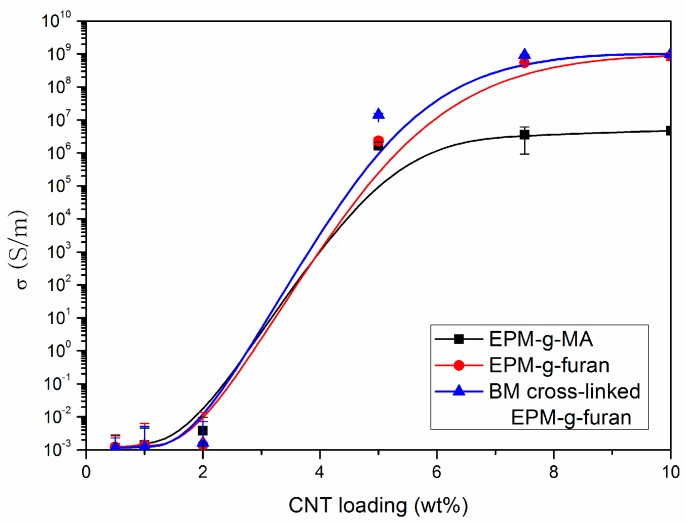
Conductivity of nanocomposites of EPM-g-MA (black), EPM-g-furan (red) or BM cross-linked EPM-g-furan (blue) with CNT as a function of the CNT loading. The error bars indicate ± 2 standard deviations.

Above a certain concentration, CNT create percolative paths in the insulating polymeric matrix in which the electrons can move with minimal resistance [35]. The percolation threshold must be studied to understand how the dispersion of the CNT affects the electrical properties of the rubber nanocomposite [36]. A substantial decrease in resistance is particularly evident for CNT concentrations close to the percolation threshold until a plateau is reached and the subsequent formation of percolation paths no longer influences the conductivity of the medium, as these do not give rise to more paths but only to alternative routes to those already existing. This large decrease in resistivity is evident in the range between 2.5% and 5% of CNT (Figure 7), which is comparable to the percolation threshold of that of other elastomeric CNT reinforced composites in the literature [2,17,34].

#### 3.2.2. Sensitivity Deformation Testing

**Figure 8:**
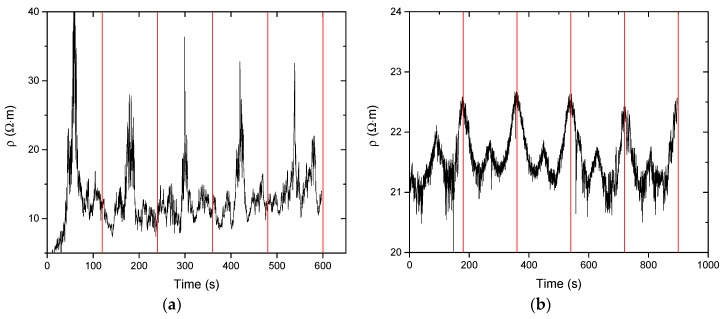
Electrical resistivity versus time over 5 cycles of 10% strain on BM cross-linked EPM-g-furan samples containing (**a**) 10 wt % and (**b**) 5 wt % of CNT. Each red line indicates the end of one cycle of strain.

Having determined the percolation threshold, the samples are used at its lower limit for low sensitivity deformation testing (high step strain with respect to high sensitivity). To illustrate the relevance of measuring samples at around their percolation threshold, high sensitivity deformation sensing measurements were performed on BM cross-linked EPM-g-furan samples containing 5 and 10 wt % of CNT with the vertical red lines indicating the end of one elongation and relaxation cycle (Figure 8). The sample containing 10 wt % CNT, i.e., well above the percolation threshold (Figure 7), displays a typical linear resistance-deformation dependence as the material reaches a maximum resistivity at full strain, that is the middle of each period. The excess of CNT in the nanocomposite allows for a large number of interconnected percolative pathways, which are gradually disrupted upon straining the sample as the CNT are pulled apart from each other. The sample containing 5 wt % of CNT, which is in the upper limit of the percolation threshold, displays non-linear resistance-deformation dependence. Unlike the previously observed “Λ”-like resistance pattern, this sample displays a “W”-like resistance pattern in each cycle of elongation and relaxation. This may be a result of the limited number of percolative pathways in this sample. The CNT that partake in these pathways are brought closer together by the tightening of the sample, orientate themselves and become more aligned by uniaxial stretching of the sample, resulting in an initial decrease in resistivity.

**Figure 9:**
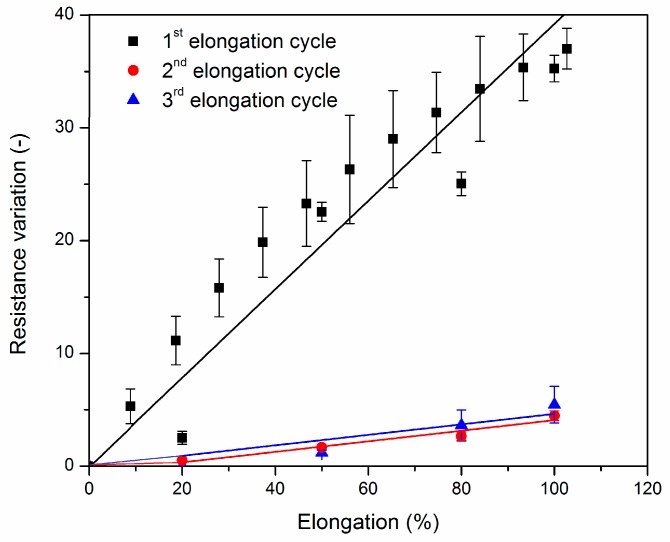
Resistance variation as a function of elongation for sample bars of BM cross-linked EPM-g-furan nanocomposites at around their percolation threshold (containing 5 wt % of CNT) for up to three cycles of elongation to 100%. The error bars indicate ± 2 standard deviations.

Non-linear resistance-deformation dependence cannot be observed in the low sensitivity experiments on BM cross-linked EPM-g-furan rubber nanocomposites containing 5 wt % of CNT (Figure 9). The reason is that these are performed stepwise instead of continuously, with large steps in strain, by which relocation of the CNT in the polymer matrix is disfavored. As expected, the electrical resistance of both samples increases gradually upon stretching because of the induced progressive breakdown of interconnected filler networks [34,35,37,38]. The relationship between the variation in resistance and the elongation represents the sensitivity of the nanocomposite as a greater variation indicates that the sensor material can detect moderate deformations. For the second and third stretching cycles of both nanocomposites, the resistance variation was therefore calculated as (*R* − *R*_0_)/*R*_0_ where *R* is the measured resistance to elongation and *R*_0_ the initial resistance of the sample.

Unfortunately, the resistance variation did not remain constant over the subsequent stretch cycles as the gauge factor decreased from 0.4 to below 0.05 after the first stretch cycle. This behavior can be attributed to the poor dispersion of the CNT (see Figure 4) and hysteresis phenomena that occur after progressive deformation cycles, scilicet once the stress is removed, the elastomeric chains gradually return to their initial state while the CNT retain the newly acquired anisotropic distribution throughout the matrix and a more effective percolation network that is maintained (larger *R*_0_) even after removal of the mechanical stress. 

### 3.3. Crack-Healing by Welding

**Figure 10:**
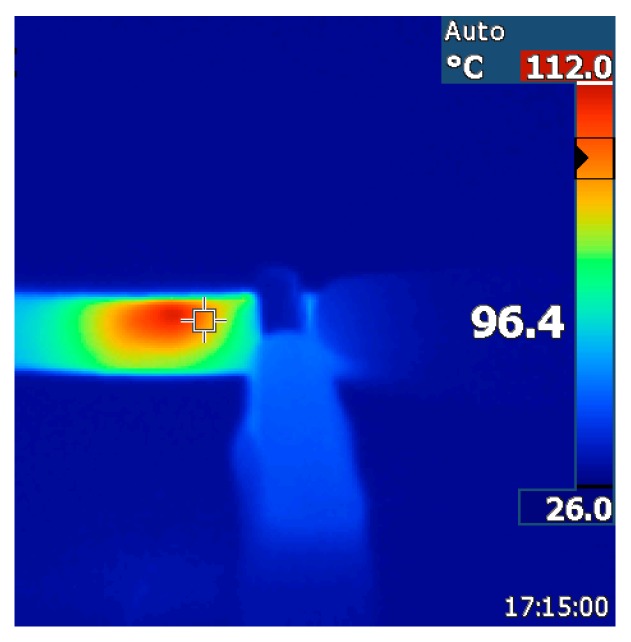
Thermal image of BM cross-linked EPM-g-furan containing 5wt % of CNT at 7 V and ~50 mA.

As the prepared rubber nanocomposites are semi-conductors, exposing them to a potential source current will result in heating up of the material via the Joule-effect [2]. This resistive heating of the sample was monitored by collecting themographic images over time (Figure 10). The effect of the increased temperature on the resistance of the material was neglected as the temperature of all tested nanocomposites reached a steady state. The dissipation of heat throughout the matrix becomes lower over time and more localized at the point of highest resistance, i.e., the interface between the CNT.

**Figure 11:**
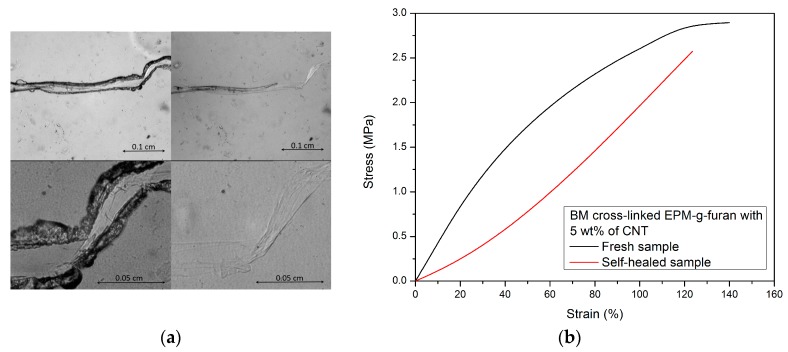
Crack-healing by welding of nancomposites of BM cross-linked EPM-g-furan with 5 wt % CNT performed by exposing the sample materials to a potential source of 7 V and 0.05. (**a**) A macroscopic scratch before and after 30 min of crack-healing by welding and (**b**) the tensile test of a nanocomposite sample bar before and after re-attaching the broken parts by crack-healing by welding for 90 min.

The temperatures observed as a consequence of autonomous heating of the materials via the Joule-effect are relatively high (>90 °C for all samples). The intrinsic temperatures at the conjugated DA cross-links may therefore be above the threshold temperature for DA re-cross-linking [39]. Crack-healing by welding due to the Joule effect [40] was therefore successfully applied to the nanocomposites at around their percolation threshold (5 wt % CNT). Microscopic scratches on the surface of the nanocomposites disappear within 30 min when exposed to a potential source of 7 V and 0.05 A (Figure 11) while broken tensile test bars would fully re-attach and regain their original tensile properties by exposing them to a current of 7 V for 90 min and compressing them in the home-made device. While the *T*_b_ and *E*_b_ of the welded sample are not different from that of the original sample bar, the shape of the stress strain curve is. This was not observed for the reprocessed samples and may therefore be due to the initial aligning of the CNT in the nanocomposite that took place during the tensile test of the original sample. This would result in an initially increased stress at relatively low strains for aligning the CNT in the original sample and a much lower stress at the same strain for the second tensile test as the CNT are already aligned.

## 4. Conclusions

In this study, conductive rubber nanocomposites were prepared by dispersing CNT in thermoreversibly cross-linked EPM-g-furan rubbers for the preparation of recyclable and reusable devices able to detect a mechanical stress via a resistive output. 

Diels-Alder chemistry was effectively used for thermoreversible cross-linking of the polymer matrix and although fully cradle-to-cradle materials were not provided, recyclable, well homogeneous, and conductive nanocomposites were obtained. Among the different strategies designed for enhancing the CNT dispersion, the rubber and the chemical modifications of the CNT appeared the most representative. An apparent increase of the cross-link density with CNT content weas obtained flanked by a high retention of tensile properties upon reprocessing. Functionalized CNT provided nanocomposites with a larger *T*_b_ and smaller *E*_b_ than with the original CNT, thus possibly suggesting their increased interaction with the polymer matrix. The percolation threshold of these nanocomposites appeared to be at a CNT loading ranging from 2.5 to 5 wt %. Low sensitivity deformation testing experiments demonstrated the necessity of using nanocomposite samples at their percolation threshold for strain-sensor applications. Only fresh samples displayed a linear response of their electrical resistivity to deformations as the resistance variation collapsed already after one cycle of elongation. This issue could be overcome by the advantages of using thermoreversible chemistry in a conductive rubber nanocomposite that were crack healed by welding due to the joule effect.

## Figures and Tables

**Table 1:** Composition and properties of a homologous series of rubber/carbon nanotube composites.

EPM * Rubber	CNT (wt %)	BM ^†^ (g)	TGA ^‡^ Residue at 600 °C (%)	Gel Content (%)	Cross-Link Density (10^−4^ mol/mL)	CNT (wt %)
EPM-g-MA	-	-	0.0	0	-	-
3.023 g EPM-g-MA	2.4	-	1.7	25	0.23	2.4
3.047 g EPM-g-MA	3.5	-	9.4	55	0.64	3.5
EPM-g-furan	-	-	0.0	0	-	-
3.019 g EPM-g-furan	2.4	-	1.6	68	0.75	2.4
3.007 g EPM-g-furan	3.4	-	9.5	82	0.95	3.4
3.010 g EPM-g-furan	-	-	0.0	93	2.1	-
3.012 g EPM-g-furan	2.4	-	1.5	95	5.2	2.4
3.036 g EPM-g-furan	4.0	0.114	4.3	96	3.6	4.0
3.024 g EPM-g-furan	4.8	0.114	4.1	98	4.0	4.8
3.146 g EPM-g-furan	5.6	0.118	10.1	99	4.7	5.6
3.028 g EPM-g-furan	6.5	0.113	7.7	99	6.3	6.5

* Ethylene propylene rubber. ^†^ Bismaleimide. ^‡^ Thermographic analysis.

**Table 2:** Tensile strength and elongation at break of EPM-g-MA, EPM-g-furan, BM cross-linked EPM-g-furan and reprocessed BM cross-linked EPM-g-furan with different amounts of (modified) CNT. Typical standard deviations ±10% and ±15–20% of the original value of the tensile strength (*T*_b_) and elongation at break (*E*_b_), respectively.

Filler Loading (wt %)		EPM-g-MA		EPM-g-Furan		BM Cross-Linked EPM-g-Furan		Reprocessed, Cross-Linked EPM-g-Furan	
		*T*_b_ (MPa)	*E*_b_ (%)	*T*_b_ (MPa)	*E*_b_ (%)	*T*_b_ (MPa)	*E*_b_ (%)	*T*_b_ (MPa)	*E*_b_ (%)
No CNT		1.2	950	1.3	750	2.2	210	1.7	190
CNT	2	2.5	250	2.5	250	3.1	200	2.9	190
	5	3.7	200	3.8	180	5.2	140	5	130
	10	5	130	5.1	130	7	80	6.5	90
modified CNT	2	2.5	240	2.6	220	3.0	210	2.9	200
	5	5.3	180	5.7	150	6.3	120	6.2	110
	10	7.2	110	7.5	100	7.8	100	7.5	90

## Appendix A

FT-IR was used to qualitatively determine the success of the modification of the CNT (Figure A1). Notably, the following bands can be detected:

- 3400 cm^−1^, characteristic of the N-H bond stretching;
- 1060 cm^−1^, characteristic of a stretching of the C–O bond;
- 1404 cm^−1^ band relating to the bending of C–O bond;
- 1399 cm^−1^ relative to the stretching of the C–N bond;
- 801 cm^−1^ band relative to the bending of the N–H bond;
- 1561 and 1560 cm^−1^ can be observed the band of the C=C stretching of the graphitic structure.

The functionalized CNT were also analysed by TGA (Figure A2). Pristine CNT are thermally stable in the presence of nitrogen up to 400–500 °C. A slight weight loss of about 2 wt % was detected above this temperature and was attributed to the more reactive portions (imperfections and structural defects) of the nanotube. The curve of functionalized CNT on the other hand, displays a clear weight loss for temperatures above 200 °C. The first derivatives of these experimental curves can be used to learn something about the nature of these weight losses.

The curve corresponding to the functionalized CNT displays a first weight loss at a temperature of 240 °C. This was attributed to a desorption of the physisorbed FFA from the nanotube walls. Conversely, weight losses observed at higher temperatures were attributed to FFA bound to the surface of the nanotube via primary interactions, such as covalent bonds. It was speculated that this loss could be preliminarily attributed to FFA intercalated in the internal cavity of the CNT. The resulting very effective interactions between the FFA and the CNT may yield a weight loss at temperatures as high as the observed 527 °C. This phenomenon is likely to occur because the internal diameters of the CNT are approximately 4 nm while the FFA molecule has an average molecular size of about 6.9 Å.

XPS evidenced the success of the functionalization process due to the presence in the photoemission spectrum of peaks attributable to the nitrogen atom at about 400 eV (Figure A3). A precise measurement of the energy of each peak provides information on the chemical state of the corresponding chemical element. The amounts of each element present in the samples were obtained from the integral of the area for each signal (Table A1). It was found that the photoemission peak of the 1s orbital of N is composed of two contributions:

- Nitrogen of the amine with an energy of 401 eV;
- Nitrogen in the form of an oxidized species (or with a different chemical environment like an adsorbed amine) with energy equal to 399 eV.

**Table A1:** The percentage of each element present in the non-functionalized and FFA functionalized CNT. Each analysis was repeated on three different portions of the sample analyzed.

	C	O	N
non-functionalized CNT	91	9	0
	90	10	0
	89	11	0
FFA functionalized CNT	93	4.9	2.0
	93	5.1	1.6
	93	5.3	1.6

SEM was performed to evaluate the morphology of the carbon nanotubes after functionalization (Figure A4). All recorded images evidenced the undamaged structure of CNT with retention of the natural flexibility of the graphitic structure and their aspect ratio that still corresponds to the size parameters given by the producer. The unaffected integrity of the CNT confirms the soft nature of the functionalization process.
